# Perils of paradigm: Complexity, policy design, and the Endocrine Disruptor Screening Program

**DOI:** 10.1186/1476-069X-4-2

**Published:** 2005-02-08

**Authors:** Jason M Vogel

**Affiliations:** 1 Environmental Studies Program, University of Colorado, Campus Box 397, Boulder, CO 80309-0397, USA

## Abstract

The Endocrine Disruptor Screening Program (EDSP), mandated by the United States Congress in the Food Quality Protection Act of 1996, attempts to protect public health from adverse endocrine effects of synthetic chemical compounds by establishing a new testing regime. But the complexities and uncertainties of endocrine disruption and its broader regulatory and social context all but ensure the failure of this policy. This article addresses the issues facing EDSP comprehensively and in detail, in order to move beyond the current regulatory paradigm and foster discourse on a positive role for scientists in support of EDSP's end goal: to protect public health.

## Introduction

The United States Environmental Protection Agency (EPA) created the Endocrine Disruptor Screening Program (EDSP) to regulate endocrine-disrupting chemicals (EDCs) as mandated in the Food Quality Protection Act of 1996 (FQPA) and the Safe Drinking Water Amendments Act of 1996 (SDWAA). Unlike the more easily appreciated effects of toxic chemicals, EDCs interact with the human body indirectly by mimicking, blocking, or otherwise disrupting the normal function of hormones. The prolific study of endocrine disruption has since uncovered many unconventional and worrisome mechanisms, exposures, and effects [1]. The goal of EDSP is to:

*[D]evelop a screening program, using appropriate validated test systems and other scientifically relevant  information, to determine whether certain substances may have an effect in humans that is similar to an effect produced by a naturally occurring estrogen, or such other endocrine effects as the [EPA] Administrator may designate *[2].

If such an effect is discovered, "the [EPA] Administrator shall, as appropriate, take action under such statutory authority as is available...as is necessary to ensure the protection of public health" [2].

Unfortunately, due to four complicating factors, EDSP cannot protect public health. The first complication is practical considerations. EPA estimates the universe of potential EDCs numbers more than 87,000 items. Testing this many chemicals would take an unreasonable investment of time and resources, but even scientifically prioritizing among them is highly problematic. The second complication is hazard complexity. Establishing relationships between EDCs and health hazards proves very difficult if not impossible. Endocrine-disrupting action breaks all the rules and assumptions that have guided toxicology through the era of modern chemical regulation. Without these simplifying assumptions, science cannot establish causation efficiently or with sufficient certainty for regulation. The third complication is exposure complexity. Determining exposure levels becomes more important and more difficult because the low-dose effects of many EDCs means that low-dose and transient exposure can be just as or more dangerous than high-dose and prolonged exposure. Assessments typically discount these ill-defined exposures, but we can no longer assume them insignificant. The final complication is regulatory deficiencies. Although FQPA and SDWAA provided new authority to test for endocrine disruption, they provided no new authority for the regulation of EDCs. As a result, multiple government agencies must manage future test-positive EDCs under their jurisdiction using fragmentary and incomplete statutory authorities and different regulatory standards. This introduces significant confusion to the institutional and decision-making aspects of the EDSP regulatory framework.

The EDSP policy design represents revision at the margins of U.S. chemical regulatory policy, not a radical revision. EDSP employs the same basic strategy used to regulate carcinogenic pesticides or toxic industrial chemicals – scientifically proving harm prior to regulating a chemical. Two important aspects of this strategy include an epistemological assumption that science has the capacity to 'prove' harm under the relevant scientific *and *legal standards, and an ethical position that prioritizes profit over human health by placing the burden of proof on public and environmental health advocates. These assumptions remain all but unchallenged in the U.S. context, and thus comprise a paradigm. While this paradigm has faced some critique in the context of carcinogenic pesticides and toxic industrial chemicals, questions of its efficacy remain unresolved. Because EDCs present new and fundamental difficulties for the science underlying the regulatory paradigm, a critical analysis of EDSP provides a more compelling case that the current chemical regulatory paradigm is in need of radical revision.

**Figure 1:**
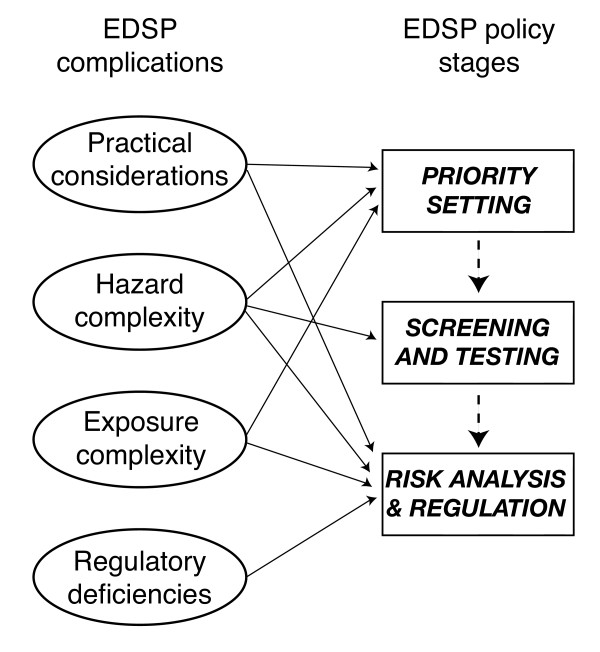
EDSP complications and policy stages relationship

This study investigates the policy design of EDSP and its broader context. The above four complications play varying roles in each stage of the EDSP policy design as discussed below. See figure 1 for a diagram roughly depicting the relationship between the four complications and the policy stages of EDSP. After considering the complications of each policy stage, this study briefly discusses the role of politics in regulation before considering the implications for the conventional chemical regulatory paradigm and a positive role for scientists in support of EDSP's end goal to protect public health.

The following discussion provides a comprehensive empirical basis for considering alternatives to the status quo. This study aims to integrate the many factors conditioning the failure of EDSP for the purpose of fostering constructive discussion on U.S. regulatory policy concerning EDCs and chemicals more generally. The author does not possess a unique answer to the many and complicated issues surrounding endocrine disruption and the U.S. chemical regulatory paradigm. Given that no simple, well-developed alternatives exist that merit immediate consideration by decision-makers, it stands to reason that more creative and open discussions of EDCs, the chemical regulatory paradigm, and possible roles for the scientific community may provide long-term payoffs in public and environmental health protection well worth our attentions today.

## Discussion

The design of EDSP consists of three main stages: priority setting, screening and testing, and a risk analysis leading to potential regulation. The complications shown in figure 1 and detailed throughout this study undermine each of these stages. Before dealing explicitly with these policy stages, however, some practical considerations deserve note. EPA estimates 87,000 chemicals require testing as potential EDCs, including pesticide chemicals, non-pesticide commercial chemicals, cosmetic ingredients, food additives, nutritional supplements, mixtures, and environmental contaminants [3,4]. This sets a daunting task; no U.S. chemical regulatory program has ever successfully tested so many chemicals. A quote from U.S. Congressman Mike Synar (D-OK), during a committee hearing on the safety of pesticides in foods states the problem dramatically: "Almost 20,000 pesticide products have been under review since 1972 and only 31 have been re-registered. At this rate it will take us to the year 15,520 A.D. to complete. I believe in good science. What I don't believe in is geologic time" [5]. Other researchers and watchdogs note the failure of other U.S. chemical regulatory programs to effectively gather information or protect public health (e.g. TSCA [6,7], and FQPA [8]; for a broader critique [9,10]). By applying Congressman Synar's analysis to endocrine-disrupting chemicals, we can expect characterization of all potential EDCs to take 59,000 years. EPA stated: "Testing of all of these chemicals cannot be supported at the same time because, even if EPA and industry had the resources to do so, there are not enough laboratories or other facilities capable of conducting the testing" [11].

In other words, there is reason and precedent to doubt our ability to accomplish this feat. More importantly, despite our best efforts to mobilize science in support of this difficult task, policy mechanisms allow chemical use and abuse to go forward regardless of scientific results or lack thereof. "Pesticides are registered for use while important health and safety data are still being generated; they may continue to be used after evidence of their hazards is given to EPA; they may be registered through alternative processes that bypass important tests; and they may never be required to be tested for certain kinds of hazards" ([9], also see [12]). Additionally, politics often plays a greater role in the decision to regulate than science (see *Politics *section below). Politics and policy design play significant roles in the modern chemical regulatory regime. Hence, a comprehensive analysis of EDC regulation must take politics and policy design as well as science into account. The appropriate standard by which to judge these disparate policy elements is Congress's mandated end goal: to protect public health. We will return to this issue.

### Priority setting

**Figure 2:**
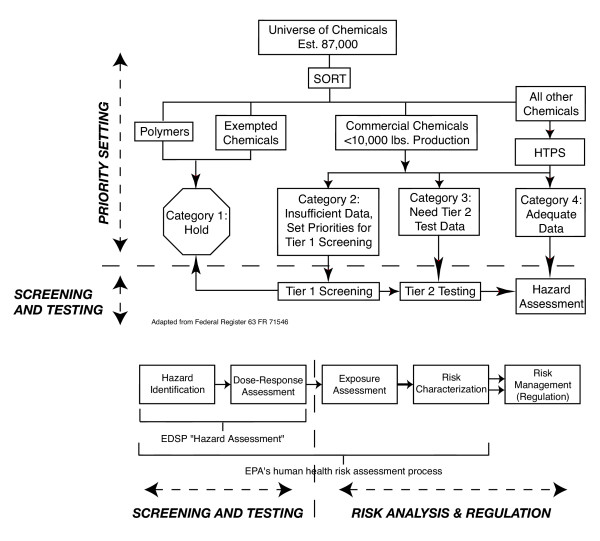
EDSP policy design

After EPA sorts chemicals according to statutory considerations, data availability, and qualitative judgment, EPA decides which of the estimated 87,000 chemicals merit consideration first through 'priority setting' [13]. The sorting of chemicals into the four categories in figure 2 and the setting of priorities within 'Category 2' require functionally equivalent information (Note figure 2 disaggregates the policy stages from the right hand column of figure 1). So priority setting, as used in this article, applies to both EDSP activities described as sorting and priority setting (i.e. everything above the dashed line in figure 2). The Endocrine Disruptor Screening and Testing Advisory Committee (EDSTAC) defined 'priority' as 'of greatest concern' in their final report, presumably as determined by science [14]. EPA, however, added statutory criteria to the scientific considerations by necessity: "EPA plans to use three main categories of information to set priorities: exposure-related information, effects-related information, and statutory criteria" [15]. Setting aside the chemicals Congress mandated EPA test (the statutory criteria), how well can EPA scientifically set priorities among potential EDCs?

EPA simply cannot set priorities based on science alone. Almost no data on potential endocrine disruption exists for the 87,000 prospective EDCs, creating a catch-22 of needing unavailable information to decide how to gather information. EPA wants to prioritize which chemicals to develop data on by examining hazard and exposure data on those chemicals. In the information poor environment of endocrine disruption, EPA has no basis to commence setting priorities. Two methodologies, high-throughput pre-screening (HTPS) and quantitative structure activity relationships (QSAR) both discussed below, have attracted attention and resources due to a general recognition of this problem. But more important than our current lack of data, EDCs operate with a high degree of complexity. Because of system complexity, some uncertainty about endocrine disruption probably cannot be resolved – resulting in some abiding doubt about the significance of a chemical as a potential endocrine disruptor. The scientific community and EPA seem quite cognizant of this complexity, but its relevance for policy and for the end goal of protecting public health deserves careful attention.

EPA has not realized their ideal of priority setting based on hazard and exposure information because of the catch-22 mentioned above. As a result, "EPA's proposed approach focuses on human exposure-related factors rather than on a combination of exposure- and [hazard]-related factors" [16]. While this statement acknowledges some of the difficulty using scientific information for priority setting, it is misleading. EPA's current stated policy prioritizes only pesticide active ingredients and high production volume (HPV) pesticidal inerts not because of scientific criteria (hazard- or exposure-related), but for statutory reasons; Congress specifically mandated testing of these compounds [2,17]. In fact, the concept of setting priorities for potential EDCs based only on exposure-related factors is fundamentally flawed.

To set priorities based on exposure factors alone, one must assume greater exposure to a chemical implies greater potential hazard (or some other arbitrary assumption). For EDCs, this assumption is scientifically insupportable. The complexity of low-dose effects (discussed in more detail in the *Screening and testing *section below) implies that exposure to some EDCs at extremely dilute doses may have a greater effect than massive exposure to that same chemical. Transient or low-concentration EDCs may also pose a greater risk than other high-exposure chemicals. Low-dose effects and other exposure complexities make exposure alone a poor proxy for setting priorities. Since different vulnerabilities and sometimes different health effects manifest at different developmental stages, any exposure-only judgment will run into significant difficulties defining spatial and temporal boundaries for exposure determinations. Some short-lived chemicals may have important endocrine- disrupting effects, but may not show up in EPA's most robust exposure data sources: biological sampling and environmental monitoring [15].

Further complexities undermine scientific determinations of exposure. Maternal metabolism of fat stores containing bioaccumulated EDCs may lead to practically unidentifiable fetal exposure. Some poorly understood exposure sources, such as flame retardants in clothing and furniture or phthalates leaching from plastics, would be extremely difficult to determine because of the complex social, cultural, and ecological conditions that affect chemical release and exposure. Even conventional exposure determinations, such as ingested pesticides, are fundamentally dependent upon patterns of food consumption. Averaging exposure may obscure vulnerabilities brought on by complicated cultural, social, and economic patterns of food consumption and other subpopulation attributes or behaviors. For example, research has shown significant differences in the exposure of adults and children to certain pesticides via residual contamination of fresh and processed foods [18,19]. FQPA may further obscure exposure determinations through mandates requiring EPA to assess cumulative exposure, including all exposure routes and sources, all chemicals with similar modes of action, and other mixtures of multiple chemicals. The complexity of endocrine disruption undermines old assumptions about the relevance of exposure and prevents scientifically meaningful prioritization on the basis of exposure data alone.

Understanding this limitation to some degree, EPA continues to develop and evaluate two methodologies to include health-effects criteria in the prioritization process. The first method is high-throughput pre-screening, or HTPS. This method allows for fast, large-scale testing of chemicals for interactions with estrogen, androgen, and now thyroid receptors. HTPS, unfortunately, has flaws as a means of detecting potential hazard for priority setting. Most basically, HTPS only tests for hormone receptor interactions. The possibility of this leading to a systematic bias against consideration of non-receptor mediated endocrine disruption is significant. Receptor interaction is only one means by which a chemical can disrupt the endocrine system. Interaction with the hormone molecules themselves, stimulation or suppression of hormone production, and disruption of old hormone metabolism can all lead to endocrine-disrupting effects as well. HTPS cannot test for these effects. Other hazard-related shortcomings relevant to HTPS are discussed more thoroughly in the *Screening and testing *section of this study. An EPA feasibility study cited some of the same issues raised here in declaring HTPS insufficient for regulatory purposes [20].

A second methodology under development is a computer modeling technique called quantitative structure activity relationships, or QSAR. QSAR simulates the behavior of a chemical based on its structure. EPA would use QSAR to predict chemical binding with estrogen, androgen, and thyroid receptors. The dominant criticism of HTPS applies to QSAR as well – it tests for receptor binding only. However, the use of computer models will incorporate new uncertainties via the selection of system boundaries and functional relationships that may preclude mechanisms and variables relevant to some endocrine-disrupting action. While a modeling effort may yield useful knowledge, as a decision-making tool QSAR is wanting. The inevitable and likely widespread false positive and false negative results will demand a parallel testing procedure to establish QSAR's utility for priority setting. But the drive to develop QSAR derives from an inability to devise an efficient and reliable testing procedure (like HTPS) in the first place. While these limitations may or may not be overcome in time, at present the methodology is not useful for setting priorities. It is instructive, however, to consider the justifications for the development of QSAR. "Systematic toxicity testing, using conventional toxicology methodologies, of single chemicals and chemical mixtures is highly impractical because of the immense numbers of chemicals and chemical mixtures involved and the limited scientific resources" [21]. QSAR was developed as an attempt to solve the very problems cited in the *Policy Design *section above. Although models can provide much useful information, they are unlikely to help prioritize EDCs anytime soon.

### Screening and testing

To amass the evidence necessary for regulation, EPA designed two tiers of scientific assays. Tier 1 screening involves short-term assays to detect potential chemical interaction with the endocrine system. Tier 2 testing involves long-term assays to establish such interactions, explore more complicated endpoints, and establish dose-response relationships. If enough data exists, a chemical can go straight to Tier 2 testing. Otherwise chemicals are assigned to Tier 1, where chemicals are prioritized and screened, with all positive results forwarded for Tier 2 testing (see figure 2 for the policy design). "The Tier 2 tests are longer in duration than Tier 1 tests and are designed to encompass critical life stages and processes as well as a broad range of doses, and are intended to be administrated by a relevant route of exposure" [16]. Although screening and testing are separate EDSP regulatory stages, their vulnerabilities to complexity are similar enough to group them together for purposes of this discussion.

Both screening and testing focus on identifying hazard, leaving exposure considerations for the final risk assessment. As such, this discussion addresses only hazard-related complexities and uncertainties. The toxicology of endocrine disruption is inherently complex in the sense that scientists must abandon the simplifying assumptions of standard toxicology. Most notably, we must abandon the assumption of monotonic dose-response relationships, which assume an increased exposure to a substance always leads to an increase in effect. Increasing exposure to some EDCs swamps the endocrine system and prevents or reduces dysfunction (i.e. an inverted U dose-response, e.g. [22,23]), while other EDCs exhibit effects at both high- and low-doses, but not in between (i.e. a U- or J-shaped dose-response, e.g. [24]); still others may exhibit hormesis, whereby a small dose has a beneficial effect [25,26].

The monotonic assumption allows for statistically significant results using smaller sample sizes exposed to higher doses for shorter periods of time. Linearly scaling these results down to typical exposure levels presumably yields approximate quantitative rates of, for example, disease or cancer. Abandoning this assumption decreases testing efficiency and multiplies the time and other resources necessary to understand the potential hazard posed by a chemical. A quote from University of Washington, Seattle toxicologist David Eaton, states the issue simply: "It's just too expensive ... you'll never be able to characterize [a low-dose effect] to the point where people think it's real" [25]. Non-monotonic dose-responses may also indicate some unresolvable system complexity. Other standard toxicological assumptions suffer the same fate as monotonic dose-response, for example: the threshold assumption and the assumption that a chemical has a uniform effect.

Other factors complicate a scientific determination of hazard. Two chemicals can interact in ways that alter their effects. Some chemicals together inhibit their individual effects, reducing or preventing an adverse effect where one is expected. Others simply add their effects together, and yet others interact synergistically, magnifying the effect either or both would normally have. "Synergistic interactions are the most problematic, because they indicate that the effects of multiple chemicals together can be significantly more powerful than might be predicted simply by adding up their effects one at a time. Regulatory science rarely incorporates any interactions; it is incapable, at present, of coping with synergies" [27]. Regardless of this incapacity, EPA seems determined to try and deal with this complexity: "EPA recognizes that the science of evaluating mixtures remains complex and unclear, but believes that it should begin to confront the issues raised by them" [28]. Additionally, scientists have evidenced possible synergism between EDCs and infectious disease agents [29]. Synergies with nutrients or poor nutrient levels might also prove significant (e.g. lead, [30]). These interaction effects further aggravate the difficulty of determining hazard. Several studies of the body burden of chemicals in humans evidence high and diverse concentrations of synthetic chemicals, indicating the importance and likelihood of chemical interactions [31-33].

Another serious complication involves the selection of testing endpoints, or dysfunctions possibly caused by EDCs. Some of the dysfunctions already identified through animal studies include cancer susceptibility and birth defects, but also more subtle endpoints like immunological dysfunction, suppression of secondary sex characteristics, decreased fertility, increased aggression, decreased mental capacity and focus, disrupted brain development, etc [34,35]. While scientists can examine some of these endpoints relatively easily in human populations (e.g. cancer incidence), others would be incredibly difficult to observe, measure, or prove with sufficient statistical certainty (e.g. feminization of boys or masculinization of girls). The difficulty of isolating and measuring these more subtle effects makes them impractical as regulatory endpoints. The inability of scientific testing to measure such endpoints, however, does not justify their exclusion from regulatory consideration. Such a policy would (and in fact does) bias the regulation of chemicals by exempting the most complicated chemicals and the most complex health effects from regulatory consideration.

### Risk analysis and regulation

A risk analysis concludes the EDSP policy design. EPA claims it will use its standard human health risk assessment process for EDCs [36]. Simply put, EPA considers hazard and exposure data and uncertainties to make regulatory decisions (see bottom figure 2 to right of dashed line). For example, an extremely hazardous chemical associated with insignificant exposure probably would not require regulation while a mildly hazardous chemical with widespread and pervasive exposure probably would. Safety factors are built into this process to protect public health. The standard safety factor for pesticides is 100× to compensate for uncertainties such as response differences between humans and the animals studied. FQPA added an additional 10× safety factor to protect children, but EPA's uses this additional safety factor inconsistently [8].

Because EPA's risk analysis demands an explicit integration of hazard and exposure data, the risk assessment itself is vulnerable to the exposure complexities treated in the *Priority setting *section and the hazard complexities treated in the *Screening and testing *section. These complexities include: HAZARD – low-dose effects, mixtures and synergies, and uncertain endpoints; and EXPOSURE – transient and low-concentration exposure to EDCs, maternal metabolism of bioaccumulated EDCs, varying vulnerability and response by developmental stage, poorly understood exposure sources, vulnerable subpopulations, and cultural, social, and economic patterns and spatial and temporal bounds of chemical release and exposure. More science cannot resolve all of these complexities and uncertainties.

But these complexities translate into an even more pernicious difficulty, as decisions assumed answerable by science must be made under conditions of scientific ambiguity. Ambiguity is related to complexity as follows: Complexity refers to properties of the system under study, not the study itself. Such properties include interactive effects (like synergies), feedback loops, temporal delays between cause and effect, chaotic or stochastic system behavior, large numbers of intervening variables, and inter-individual variations. Uncertainty refers to limitations of the human analysis of a complex system. While science is often employed 'to reduce uncertainty,' the complexity of a system sets bounds on the prospective certainty of informed scientific judgment. For example, further science can never remove the chaos from a chaotic system or the randomness from a stochastic one. And to illustrate the point, exposure of a fetus to environmental EDC contamination *in utero *might easily be a chaotic or stochastic system. Ambiguity refers to a situation in which existing scientific data support equally valid but competing interpretations of risk (see [37] for a discussion of concepts).

Ambiguity is the single greatest limitation to the use of endocrine disruption science in policy. While an oft-cited truism holds that decision-makers can make better decisions with reliable information at hand, this hardly tells the whole story. Other considerations play into decision-making, including value tradeoffs, time and resource limitations, and informational constraints, including scientific ambiguity. A National Academy of Sciences report on endocrine disruption substantiates the ambiguity in this field:

*[I]t became clear as the work of the committee progressed that the same data could be approached from different viewpoints. Those different views led to different judgments among the committee members about the significance of the threat posed by [EDCs]. In [some] cases, the differences do not reflect the need for research but reflect differing judgments about the significance of information. The differences are not confined to the members of this committee but are also reflected in the scientific community at large and in the comments received during review *[35].

The recommendations of this committee amounted to suggestions for more research. While more research might be a good thing, such a suggestion provides no guidance on how to address the more policy-relevant question of how to make decisions with ambiguous scientific information. In a nutshell, more science is not a panacea for all the problems of risk analysis or decision-making.

This brings us to making decisions about regulation. If a reasonably certain determination of harm about an EDC could somehow be made, how would one regulate that chemical? This discussion of regulation is only tentative since EPA's Regulatory Activities Workgroup continues to study the issue. Since EPA has not yet validated any screening or testing procedures [38], regulation under EDSP has so far received little attention. Under section 408p of FQPA, EPA must use "such statutory authority as is *available*" to protect public health [2] (emphasis added). In other words, the statutes requiring testing for endocrine disruption provide no new process by which to regulate those chemicals – the standards for regulation remain those of previous regulatory laws. Unfortunately, this complicates enforcement authority for EDCs.

Regulation must be authorized under one of four laws: the Toxic Substances Control Act (TSCA), the Federal Food, Drug, and Cosmetic Act (FFDCA), the Federal Insecticide, Fungicide, and Rodenticide Act (FIFRA), or the Safe Drinking Water Act (SDWA). The agencies with regulatory jurisdiction for EPA's list of 87,000 chemicals are: the Environmental Protection Agency (EPA), the Food and Drug Administration (FDA) in the U.S. Department of Health and Human Services, and the Food Safety and Inspection Service (FSIS) in the U.S. Department of Agriculture. EDSTAC recommended and EPA adopted the following list of chemicals for endocrine disruptor testing: 75,500 commercial chemicals listed under TSCA, 900 pesticide active ingredients, 2,500 pesticide inert ingredients, 5,000 cosmetic ingredients, 3,000 food additives, an unspecified number of nutritional substances, and an unspecified number of natural hormonally active plant residues [14].

Testing and enforcement authority for this universe of chemicals is fragmentary. For example, EPA has authority under FIFRA and FFDCA to set tolerances for pesticides on food, but enforcement authority falls to FSIS for meat and poultry products, and FDA for other food items. The authority is also incomplete. For example, FDA has authority over the estimated 5,000 cosmetic chemicals, but FDA has no authority to require any information from the manufacturer or to mandate product safety testing. FDA's regulatory authority over cosmetics begins only after a product (possibly without any safety information) enters the market. Additionally, the standards by which to regulate differ. Under TSCA and SDWA the economic costs of regulation must be balanced against the public health threat, but under FIFRA and FFDCA, economics can be considered in only narrowly crafted situations – the standard is largely health-only based. FFDCA and FIFRA as amended by FQPA use the "reasonable certainty of no harm" standard. This standard translates into a 95% certainty that fewer than one in a million additional cancer deaths will occur due to the expected exposure over a lifetime. No translation specific to EDCs of this standard is yet available. TSCA, on the other hand, must prevent "unreasonable risk" of injury to health or the environment. A risk is "unreasonable" if the risks exceed the benefits associated with that activity, including economic benefits. SDWA explicitly requires a consideration of the cost of compliance to state, local, and other water systems when setting safety standards.

The estimated 2,500 pesticide inert ingredients may defy regulation due to trade secret norms, poor EPA data quality, and historic government neglect [39,40]. Additionally, no federal statute delegates any authority at all for the testing or regulation of nutritional supplements. Presumably, endocrine-disrupting nutritional supplements could be regulated only voluntarily, but the onus of testing would fall completely upon the executive agency that volunteers to expand its responsibilities. Significant difficulties involving confidential business information, including proprietary chemicals and chemical mixtures may further compromise enforcement capability.

The complexity of the institutional and legal landscape (multiple interacting agencies with multiple overlapping mandates and authority) creates substantial regulatory confusion. In this situation, the powers and responsibilities of different government agencies might be interpreted differently by other agencies or by affected parties. Such confusion leaves room for interpretation that may require long delays and intensive court battles to resolve. The history of TSCA indicates that such confusion (for TSCA, 'balancing economic cost' with regulations to protect public health) as well as the menace of legal action can lead to crippling regulatory inaction [41]. By requiring enforcement under existing statutory authority, FQPA leaves the regulation of the already complicated universe of EDCs to a complicated web of regulatory regimes of questionable efficacy.

## Politics

Finally, the role of politics in regulatory decision-making deserves note. The conventional ideal of regulation under the current paradigm is that good science leads to an informed decision-maker who can then remove or limit a proven hazardous chemical from commerce (or, rarely, prevent its introduction). The complexities of endocrine disruption science, practical considerations, and the regulatory deficiencies discussed above impose limitations on this conventional ideal. Neglecting the role of politics in regulatory decision-making, however, is perhaps this ideal's most significant omission. A variety of academic and government research as well as environmental and public interest group analysis points to the failure of testing regimes to produce significant regulation or protect public health (e.g. [7,8,10,12,41,42]). In fact, past regulation often addressed specific chemicals by legislative mandate (e.g. the mandated ban of PCBs in TSCA) or due to media-promoted public awareness and its resultant outcry (e.g. the January 1971 court order essentially forcing EPA Administrator Ruckelshaus to ban DDT). In other words, politics often leads to regulation regardless of scientific considerations.

The Alar 'scare' of 1989, when the public and EPA reacted to evidence that contamination of apples might endanger child health, provides a visible recent example of this dynamic. A *60 Minutes *show aired on February 26, 1989 [43] dedicated to the findings of a Natural Resources Defense Council study titled 'Intolerable Risk: Pesticides in our Children's Food' [18]. The public outcry about Alar (a.k.a. daminozide) led to a drop in apple sales and pushed EPA and Alar's manufacturer, Uniroyal Chemical Company, Inc., to take action [44]. After announcing the safety of Alar in March 1989, and an intention to take no action before July 1990 [45], EPA announced a preliminary determination to eventually cancel all registrations of daminozide used on foods in May 1989 [46]. A little over a week later, Uniroyal announced a voluntary recall of all remaining stocks of Alar, and EPA approved a voluntary cancellation of all Uniroyal's daminozide registrations that November [47].

In an attempt to avoid the still bitter battle between Alar critics and advocates, the relevant point is not whether Alar is or is not a health hazard, but that politics played a major if not a dominant role in its regulation. The very fact that a bitter argument about the actual risk posed by Alar persists indicates that science does not always play a definitive role in regulatory decision-making [44,48,49]. But if politics significantly affects decision-making, what is the role of science? To understand the interplay between science and regulation, we must critically consider the conventional assumptions of the modern U.S. chemical regulatory paradigm.

## Conclusions

The conventional paradigm underlying EDSP and most other U.S. chemical regulation amounts to 'science leads to regulation;' it assumes a scientific determination of harm must and, in fact, does precede regulatory action. In this context, Congress mandated EPA protect public health from EDCs, but only *after *"develop[ing] a screening program, using appropriate validated test systems and other scientifically relevant information, to determine [harm]" [2]. Real progress on protecting public health waits on the development of a scientific testing regime, on faith that scientific testing is both necessary and sufficient to protect public health. Practical considerations, hazard complexity, exposure complexity, and regulatory deficiencies all challenge the naivety of this assumption. Rational analysis of these factors leads to the inescapable conclusion that science has limitations within the existing regulatory regime and that other important factors are disregarded by the current paradigm.

This criticism does not discount the contributions of science, although it seriously questions the assumption that simply doing more science will protect public health. Additionally, this argument does not promote unconsidered action, although it does stress the need to make decisions in the face of ambiguous information. The paradigm itself, though invisible to most adherents, is quite real. The words and actions of industry and environmental groups, government agency personnel, members of Congress, and other concerned interests, regardless of their side of the debate, indicate near universal buy-in to the 'science leads to regulation' paradigm (see [10] for discussion). But the complexity, uncertainty, and ambiguity of endocrine disruption and its broader context undermine this paradigm's simple logic.

The internal validity of 'science leads to regulation' presumes the capacity of science to prove harm with sufficient certainty to regulate. Exposure complexities, including transient and low-concentration exposure to EDCs, maternal metabolism of bioaccumulated EDCs, varying vulnerability and response by developmental stage, poorly understood exposure sources, vulnerable subpopulations, and cultural, social, and economic patterns and spatial and temporal bounds of chemical release and exposure, place the ability of science to make solid exposure determinations in significant doubt (see *Priority setting *section). Hazard complexities, including low-dose effects, mixtures and synergies, and uncertain endpoints, contribute further obstacles to an unambiguous scientific determination of harm (see *Screening and testing *section). Under current policy mechanisms, these complications of endocrine-disruption science will prevent any meaningful regulatory action. Essentially, endocrine disruption is too complex and our science too uncertain; most scientific information regarding EDCs will remain ambiguous, with the available information supporting quite different judgments of risk.

Recall this observation by the National Research Council: "In [some] cases, the differences [in scientific judgments of EDC significance] do not reflect the need for research but reflect differing judgments about the significance of information" [35]. The National Research Council has also recognized the more general prejudice that hinders regulation: "The assumption of the null hypothesis as used in risk analysis [as in the case of regulating chemicals] contains an implicit bias because it places a greater burden of proof on those who would restrict than those who would pursue a hazardous activity, presuming these activities are safe until proven otherwise" [50]. In other words, the paradigm is biased against regulation, and the complexity and uncertainty of endocrine disruption will further undermine attempts to regulate.

The external validity of 'science leads to regulation' must take into account the broader context of potential endocrine disruptor regulation. The regulatory deficiencies addressed in the *Risk analysis and regulation *section, the practical considerations addressed in the *Policy Design *section, and the significant role of politics in regulation discussed in the *Politics *section challenge the arbitrary constraints the paradigm places on non-scientific factors. The roles of actors besides the scientific community and agency scientists can make or break regulation. Furthermore, the legal ambiguity of regulating different chemicals under four statutes with different regulatory standards and fragmentary, incomplete statutory authority guarantees further difficulty with regulation, even if the science could meet the near-impossible burden of proof. The most basic practical considerations, including other U.S. regulatory precedents, policy mechanisms to avoid regulation, and most importantly, the sheer number of potential EDCs, each bring the conventional paradigm into doubt. 'Science leads to regulation' simply leaves too much of the decision-making context out of the picture.

Yet discussions of improving chemical regulation primarily deal with the minutiae of scientific testing regimes. For example, an Environmental Defense Fund analysis exposing the utter futility of chemical regulation under TSCA (based on the U.S. Government's own damning analysis) came to the conclusion that the failure of testing in the past means we need to test better in the future [7,41]. Though better testing might improve things, such a suggestion still ignores the hard reality of decision-making. Scientific information often remains ambiguous and consistent with quite different action alternatives. Furthermore, scientific information forever remains only one consideration of the decision maker – economic impact, resource tradeoffs, political considerations, constituent needs, and agency funding are other obvious and equally relevant considerations.

The relevance of science for regulatory decision-making lies predominantly outside the current trend of increasingly detailed mechanistic investigation of endocrine disruption. While endocrine disruption science can contribute much to a decision-maker, it cannot provide unambiguous information that 'objectively' determines the correct decision. But science could help guide decision-making under conditions of ambiguity; uncertainty does not entail 'anything goes.' On the contrary, the uncertainty itself might guide decision-making better than any other factual information. The 2001 Intergovernmental Panel on Climate Change reports provide a precedent in the explicit treatment of uncertainty – the reports indicate the relevance of scientific information for decision makers by ranking scientific conclusions by Bayesian confidence estimates (e.g. virtually certain means greater than 99% confidence, very likely 90–99%, etc) [51]. This empowers decision makers and the public by contextualizing expert knowledge and frees scientists to provide relevant information that has not yet met the rigorous standards of scientific proof and peer review. Some excellent work extrapolating from this precedent and considering its ramifications already exists [52,53].

A franker treatment of uncertainty can improve the relevance of science to decision-making and promote realistic expectations of science and the scientific community. Such progress might turn the spotlight on more significant impediments to regulatory decision-making under conditions of ambiguity, such as the difficulty of testing 87,000 chemicals and the role of politics and values in making regulatory decisions. Scientists can take action within their own communities to support the policy goal of protecting public health by exploiting and improving the role scientific information plays in chemical regulation. The possibility that scientists can empower decision makers and the public by developing community standards and norms explicitly addressing uncertainty is one creative idea, but more are needed. The key to progress is taking an active and creative role in support of the protection of public health. Waiting for a solution in the form of national legislation has failed to inspire significant change over the last several decades. But explicit action within the scientific community that discourages unrealistic expectations of science will support long-term progress on chemical regulatory policy and the protection of public health.

## List of abbreviations

DDT – dichlorodiphenyltrichloroethane

EDC(s) – endocrine disrupting chemical(s)

EDSP – (U.S.) Endocrine Disruptor Screening Program

EDSTAC – Endocrine Disruptor Screening and Testing Advisory Committee

EPA – (U.S.) Environmental Protection Agency

FDA – (U.S.) Food and Drug Administration

FFDCA – (U.S.) Federal Food, Drug, and Cosmetic Act

FIFRA – (U.S.) Federal Insecticide, Fungicide, and Rodenticide Act

FSIS – (U.S.) Food Safety and Inspection Service

FQPA – (U.S.) Food Quality Protection Act

HPV – high production volume

HTPS – high-throughput pre-screening

PCBs – polychlorinated biphenyls

QSAR – quantitative structure activity relationships

SDWA – (U.S.) Safe Drinking Water Act

SDWAA – (U.S.) Safe Drinking Water Amendments Act

TSCA – (U.S.) Toxic Substances Control Act

## Competing interests

The author(s) declare that they have no competing interests.
